# A nosocomial transmission of crimean-congo hemorrhagic fever to an attending physician in north kordufan, Sudan

**DOI:** 10.1186/1743-422X-8-303

**Published:** 2011-06-15

**Authors:** Afraa T Elata, Mubarak S Karsany, Rehab M Elageb, Marwa A Hussain, Kamal H Eltom, Mustafa I Elbashir, Imadeldin E Aradaib

**Affiliations:** 1 Molecular Biology Laboratory (MBL), Department of Clinical Medicine, Faculty of Veterinary Medicine, University of Khartoum, P.O. Box 32 Khartoum North, Sudan; 2 Division of Virology, National Public Health Laboratory, Federal Ministry of Health, Republic of the Sudan; 3 Department of Biochemistry, Faculty of Medicine, University of Khartoum, Sudan

## Abstract

**Background:**

Crimean-Congo hemorrhagic fever (CCHF), a tick-borne disease caused by Crimean-Congo hemorrhagic fever virus (CCHFV), is a member of the genus Nairovirus in the family Bunyaviridae. Recently, CCHFV has been reported as an important emerging infectious viral pathogen in Sudan. Sporadic cases and multiple CCHF outbreaks, associated with nosocomial chain of transmission, have been reported in the Kordufan region of Sudan.

**Aims:**

To confirm CCHF in an index patient and attending physician in North Kordufan region, Sudan, and to provide some information on virus genetic lineages.

**Methods:**

Antibody captured ELISA, reverse transcription PCR, partial S segment sequences of the virus and subsequent phylogenetic analysis were used to confirm the CCHFV infection and to determine the virus genetic lineages.

**Results:**

CCHF was confirmed by monitoring specific IgM antibody and by detection of the viral genome using RT-PCR. Treatment with oral ribavirin, replacement with fluid therapy, blood transfusion and administration of platelets concentrate resulted in rapid improvement of the health condition of the female physician. Phylogenetic analysis of the partial S segment sequences of the 2 CCHFV indicates that both strains are identical and belong to Group III virus lineage, which includes viruses from Africa including, Sudan, Mauritania, South Africa and Nigeria.

**Conclusion:**

Further epidemiologic studies including, CCHFV complete genome analysis and implementation of improved surveillance are urgently needed to better predict and respond to CCHF outbreaks in the Kordufan region, Sudan.

## Introduction

Crimean-Congo hemorrhagic fever (CCHF), caused by Crimean-Congo hemorrhagic fever virus (CCHFV), is a viral zoonotic disease with a high mortality rate in humans. CCHFV can be transmitted to humans by bites of *Ixodid *ticks or by contact with blood, bodily fluids or tissue from viraemic livestock and infected humans [1]. CCHFV is one of the rare hemorrhagic fever viruses capable of inducing nosocomial outbreaks in hospitals with resource-poor setting and person to person transmission is not uncommon [2,3]. CCHF is a public health problem in many regions of the world including the Sudan [4-13]. In remote areas and rural hospitals of Kordufan, Sudan, diagnosis of the disease is primarily based on clinical presentation. The laboratory diagnosis of CCHF includes the serological tests for the detection of immunoglobulin M and immunoglobulin G antibodies and molecular-based technique such as conventional and real-time RT-PCR for detection of the viral genome [5,14,15].

In the past few years, CCHFV has been reported as an important emerging infectious viral pathogen in Sudan. We reported on nosocomial outbreak of CCHF in Alfulah Rural Hospital, West Kordufan. Two virus strains designated Al-fulah 3 and 4 (GenBank accession Nos.GQ862371-2) were identified as etiologic agents of the nosocomial outbreak [4]. We have also reported on an outbreak in Donkup village, Abyei District, South Kordufan [5]. Despite the fact that the Alfulah and Abyei strains belong to group III genetic lineage of CCHFV they are genetically distinct from each other and were identified as unique strains of the Sudanese CCHFV. Abyei and Alfulah virus strains are considered to be responsible for the emergence of the disease in Western and Southern region of Kordufan [4,5]. However, CCHF has never been reported in North Kordufan.

In February, 2010, an index patient from Lagawa District, Southern Kordufan, Sudan, was admitted to Lagawa Rural Hospital, with an acute hemorrhagic illness. Lagawa District is approximately 50 Kilometers from Alfulah, the origin of the first CCHF outbreak in Sudan. The index patient is a 60-years-old male who was admitted with clinical presentation of an acute febrile hemorrhagic illness. The symptoms included rapid onset of fever, headache, nausea, vomiting of blood, and bloody diarrhea. The source of infection was suspected to have been the result of consumption of raw liver of an infected sheep. He complained of high grade fever, chills, headache, epistaxis, vomiting of blood and bloody diarrhea. He had taken anti-malarial medication at home without improvement. The patient was then transferred to Kadogli Hospital, the referral hospital for the State of Southern Kordufan, approximately 200 kilometers from Lagawa District. As the medical facilities in Kadogli Hospital are limited, his health condition deteriorated very rapidly and thus the patient sought additional health care at Elobied Hospital, the capital of North Kordufan State. At Elobied Hospital, the patient was subjected to medical examination by the attending physician on Saturday, 20/02/2010 and was provided medical care by nurse. Clinical investigations were conducted and blood samples were collected after which the patient was referred to Khartoum for further medical care. The patient was taken care of at the hospital and he survived the infection and discharged from the hospital in a good health.

On Tuesday, 23/02/2010, the attending physician developed high grade fever, backache, vertigo and she became hypoglycemic. She received medical care and nursing by her mother and sister. A few days later, her sister had onset of a disease which did not progress beyond mild fever. Blood films from the index patient and the physician were malaria positive with high parasitemia. Treatment with an antimalarial drug did not result in any improvement. Thus, the attending physician was isolated and strict barrier nursing was implemented on the suspicion of viral hemorrhagic fever (VHF). On Saturday 27/02/2010, the health condition of the physician deteriorated very rapidly and she developed massive vaginal bleeding, which lasted after 12 days. She also developed renal insufficiency. The serum urea and creatinine levels were as high as 135 mg/dL and 13 mg/dL, respectively. Blood platelets count was as low as 9 x10^3^/μl. Treatment with oral ribavirin at a dose rate of 500 mg 4 times a day for 5 days, compensation with four vials of blood and administration of platelets concentrate resulted in rapid improvement of her health condition one week later. The blood platelets counts increased dramatically to 169 × 10^3 ^/μl. She was discharged from the hospital on Tuesday 16/3/2010 in a good health condition with platelets count of 252 × 10^3 ^/μl.

In the present investigation, we report on a nosocomial transmission of CCHF to an attending physician in North Kordufan as a result of medical referral of an infected patient, who is resident of Lagawa District, South Kordufan. Rapid diagnosis allows proper management of the disease during outbreak settings. In addition, molecular characterization of the virus provides information on the virus genetic lineages, which subsequently provides a more detailed understanding of the movement of virus strains in Kordufan region and in Sudan at large [4].

## Materials and methods

### Crimean Congo hemorrhagic fever Case Definition

The case definition used for identification of Crimean Congo hemorrhagic fever patients included hemorrhagic manifestations (ecchymosis, petechia, epistaxis, hematemesis, and hemorrhagic enteritis as reflected by bloody diarrhoea), and one or more of the following symptoms: Fever, severe headache, joint pain, chills, and nausea.

### Ethical clearance and informed consent

Blood samples were collected from the two acute hemorrhagic fever (HF) patients in clean, sterile vacutainers. Samples were collected as part of routine diagnostic testing. Ethical clearance was obtained from the Ministry of Health of Sudan and informed consent from all patients was provided through an ethical clearance form, which permitted use of the samples for diagnostic and research purposes. In addition to being used for routine malaria screening, blood was allowed to clot, and sera were separated and sent to the Division of Virology, National Medical Health Laboratory, Khartoum, Sudan, for serological diagnostic screening. Extracted RNAs from serum samples were provided to the Molecular Biology Laboratory, Faculty of Veterinary Medicine, University of Khartoum for conventional RT-PCR amplification and subsequent sequencing.

### Serology and Molecular Diagnostic

Virus isolation attempts were not conducted as the cultivation of CCHFV requires high laboratory containments (biosafety level 4), a facility which is restricted to a few laboratories in Africa. Therefore, identification of the virus was solely dependent on serology and conventional RT-PCR amplification, using primers targeting the S segment of the Al-fulah strain of CCHFV (Genbank accession number GQ862371), followed by direct sequencing of the PCR ampilicon. Serologic tests were conducted to screen the sera for CCHFV antibodies as determined by detection of IgM using antibody capture enzyme-linked immunosorbent assay (ELISA) kits from Biological Diagnostic Supplies Limited (South Africa).

### Design of primers

It is well documented that CCHFV small (S) RNA genome segment, which codes for viral nucleoprotein (NP) and non-structural protein, has the less variable nucleotide sequences among cognate genes of CCHFV strains [16]. Thus, selection of the primers was based on a highly conserved fragment of the S RNA segment of Sudan, Alfulah-4 strain (GenBank accession number GQ862371). The primers were designed based on multiple sequence alignment of several published sequences of the gene using BioEidit software (Carlsbad, CA, USA). A forward primer CCHF1 (5): CTG CTC TGG TGG AGG CAA CAA (3) and a reverse primer CCHF2 (5): TGG GTT GAA GGC CAT GAT GTA T (3) were used to amplify a 452-bp primary PCR product. An internal pair of forward primer CCHFn1 (5): AGG TTT CCG TGT CAA TGC AAA (3) and a reverse primer CCHFn2 (5): TTG ACA AAC TCC CTG CAC CAG T (3) were used to amplify a 207-bp nested PCR product.

### Reverse transcription (RT) Polymerase chain reaction (RT-PCR) amplification

A single-tube RT-PCR assay was carried out for CCHFV RNAs amplification basically as described previously [17]. Briefly, a standard 50 μl reaction mixture contained in final concentration of 1 × enzyme mix reaction buffer, 5.0 μl of 10 mM dNTP mix, 5.0 μl of 25 mM Mg Cl_2_, 5.0 U enzyme mix, 2.0 μl of 20 picomole of each primers (CCHF1 and CCHF2), 5.0 μl of target RNA, were used. The total volume was brought to 50.0 μl using RNase free water. Target genes were amplified in low-profile 0.2 ml tube (MJ Research, California, USA). Rift Valley fever virus (RVFV) and Dengue virus RNA templates were used as negative controls. Thermal profiles were performed on a Techne PHC-2 thermal cycler (Techne, Princeton, NJ). The thermal cycling profiles were started with 30-min incubation at 50°C for reverse transcription of the CCHFV RNA templates into cDNA copies. The PCR tubes were then incubated at 95°C for 15 min to destroy the excess amount of RT enzyme and to activate the Taq DNA polymerase. The synthesized cDNA copies in the PCR tubes were subjected to 40 cycles of denaturation at 95°C for 1 min, annealing at 56°C for 30 sec and extension at 72°C for 45 sec, and a final incubation at 72°C for 10 min.

### Nested RT-PCR amplification

For nested amplification, reaction mixtures similar to those of RT-PCR were used, except that 2 μl of the primary PCR products were used as a template DNA for PCR amplification. The thermal cycling profiles were started with an initial denaturation at 95°C for 5-min, and the PCR tubes were subjected to 40 cycles of denaturation at 95°C for 1 min, annealing at 56°C for 30 sec and extension at 72°C for 45 sec, and a final incubation at 72°C for 10 min. Following amplification, 10 μl from each PCR amplification product were loaded onto 2% agarose gel and electrophoresed at 80 volts for 1 h. The gels were stained with ethidium bromide, and a UV light source was used to visualize the primary and the nested PCR products.

### Sequence analysis and phylogenetic relationship

The 452-bp primary PCR products were purified using QIAquick PCR purification Kit (QIAgen, Germany) and sent for sequencing in a commercial company (Seqlab, Göttingen, Germany). Resulted sequences were edited using BioEdit software and the Basic Local Alignment Search Tool (BLAST) of NCBI (National Center for Biotechnology Information, Bethesda, MD) and used to confirm the identity of the generated sequences in the GenBank nucleotide database. The sequences were then aligned with the corresponding S-segment of known CCHFV strains from Sudan, other African countries, Asia, middle and far east, and Eastern Europe. Phylogenetic tree was constructed using PAUP version 4.0 software packgae (Sinauer Associates, Inc., Sunderland, MA, USA). The name of the virus strain, country of origin and the GenBank accession numbers were given for each sequence.

## Results

### Serologic studies

In the present study, serological attempts to diagnose CCHF in acute phase sera sampled during the first 3 days post-infection (pi) from both patients were largely unsuccessful. However, positive IgM antibodies to CCHFV were detected in subsequent blood samples drawn from the attending physician at day 7 pi.

### Reverse-transcription (RT) polymerase chain reaction (RT-PCR)

**Figure 1:**
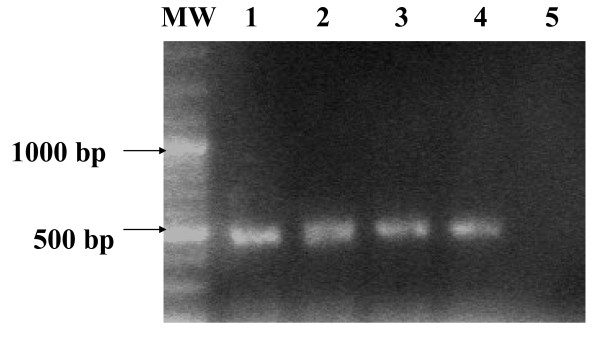
**Visualization of the primary 452-bp CCHFV PCR product on ethidium bromide-stained agarose gel from 1.0 pg of RNA of Sudanese isolates of CCHFV variants**. Lane MW: molecular weight marker (100 bp DNA ladder); Lane 1: Alfulah strain (positive control); Lane 2: Abyei strain; Lane 3-4: RNA extracted from sera of the index patient and the attending physician (Lagawa strains); Lane 5: RNA extracted from sera of healthy non infected person.

**Figure 2:**
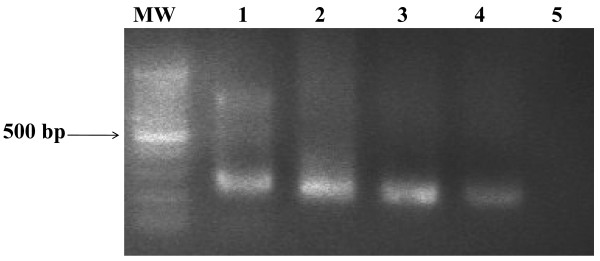
Visualization of the nested 207-bp PCR products from the primary 452-bp PCR products

**Figure 3:**
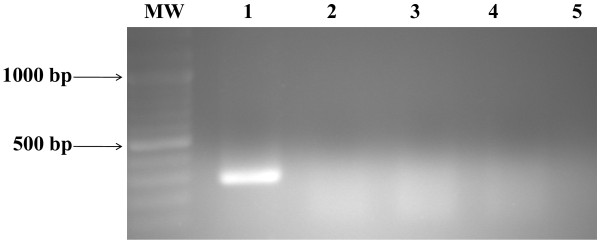
**Specificity of the nested CCHFV RT-PCR for detection of CCHFV RNA**. Lane MW: molecular weight marker (100 bp DNA ladder); Lane 1: Alfulah strain (positive control); Lane 2: RVFV RNA; Lane 3: Dengue virus RNA; Lane 4-5: RNA extracted from sera of healthy non infected persons.

Application of the reverse transcriptase-polymerase chain reaction (RT-PCR) assay, to acute phase sera sampled during the first 3 days pi from both suspected HF patients resulted in amplification of a primary 452 bp PCR product (Figure 1). The second round of nested RT-PCR amplification produced a 207 bp PCR product specific for CCHFV S segment (Figure 2). No amplification products were detected for RNAs from closely related viral hemorrhagic fever viruses including Rift valley fever virus (RVFV), Dengue virus, total nucleic acid extracts from uninfected Vero cells, and sera from non- infected patients (Figure 3).

### Sequence analysis and phylogenetic relationship

**Figure 4:**
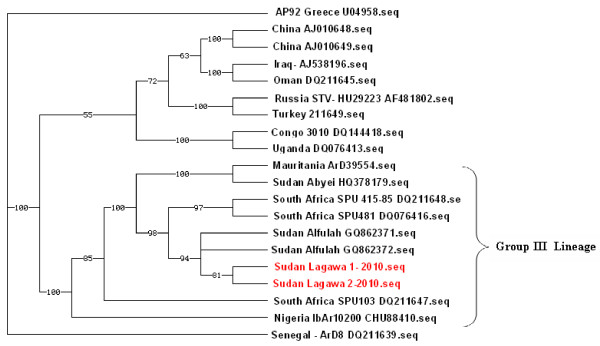
**Phylogenetic relationship of Sudan-Lagawa strains of CCHFV**. Partial S segment sequences of the virus strains were aligned with other CCHFV strains from Sudan, different African regions, the Middle and Far East, Asia, and Eastern Europe. Sequeces were analyzed with the Lasergene 3.57 software package (DNASTAR, Madison, WI, USA). Phylogenetic tree was constructed using PAUP version 4.0 (Sinauer Associates, Inc., Sunderland, MA, USA). Bootstrap values were calculated from analysis of 500 replicates of the data set, and values greater than 50% are indicated at the appropriate nodes. Each CCHFV sequence is designated by the name of the strain, country of origin. The GenBank accession numbers are given for each CCHFV isolate. The two Lagawa strains from Sudan (Sudan Lagawa1 and 2-2010) are highlighted in red color for clarity (GenBank accession number (HG829854). Brace indicates group III virus genetic lineage (16).

The sequences from the primary 452-bp PCR products were determined by BLAST/FASTA (http://www.ncbi.nlm.nih.gov) and phylogenetic analysis. The sequences were edited and assembled using Lasergene software 3.57 (DNASTAR, Madison, WI, USA). The partial S-segment of Lagawa strain showed 99% homology with Sudan- Alfulah strain-3. Phylogenetic tree was constructed using PAUP version 4.0 software (Sinauer Associates, Inc., Sunderland, MA, USA) to investigate the relationship between Lagawa CCHFV strain and other known CCHFV strains identified globally. The phylogenetic analysis placed this virus strain in Group III virus lineage, which includes virus strains from across Africa including Sudan, Mauritania, South Africa and Nigeria (Figure 4). The partial S-segment sequence was submitted to the GenBank under accession number HQ829854.

## Discussion

Multiple Crimean-Congo hemorrhagic fever virus (CCHFV) variants and strains, associated with CCHF outbreaks, have recently been reported in Alfulah, Western Kordufan [4] and in Abyei, Southern Kordufan of Sudan [5]. However, CCHF has never been reported in North Kordufan. In this study, a nosocomial transmission of CCHF has been reported in an attending physician in North Kordufan as a result of medical referral of an infected patient from an endemic area in Lagawa District, South Kordufan. In the present study, rapid diagnosis of the disease was achieved using conventional nested RT-PCR. The nested RT-PCR assay could be used for detection of CCHFV in a simple laboratory setting. In addition, the assay detected the multiple CCHFV strains identified, so far, in Sudan. The first RT-PCR round of amplification would be sufficient for detection of CCHFV in sera sampled during acute phase of the disease, as the titer of the virus is usually high during this period, which allows for molecular characterization studies without initial amplification of the virus in cell culture. However, the second round of nested amplification may only be required, at later stage of the disease, when the concentration of the virus in sera or tissue samples is very low. The well characterized primers designed from a highly conserved region of the S-segment RNA would be expected to detect the African CCHFV variants in group III virus lineage and would probably detect the remaining CCHFV from other virus lineages.

The source of the infection of the index patient was considered to be the result of consumption of raw sheep liver. In certain areas of Sudan, the social habit of consumption of raw offals of cattle and raw livers of sheep with bile and spices is not uncommon, which serves as a means of transmission of various communicable diseases [18] and CCHF is not an exception. It is noteworthy that seasonality for CCHFV infection is quite evident and usually coincides with the influx of sheep from rural areas and villages to towns and major cities on certain religious occasions [2]. Under these circumstances, the animal attendants typically live in close contact with their animals. This will increase the risk of acquiring CCHFV infection through direct contact with infected animals or ticks.

Taking into consideration the identification of multiple virus variants and strains in human cases from Alfulah (2010) and Abyei districts of Kordufan region (2011), in addition to the two CCHF cases mentioned in the present study, it is likely that these CCHFV strains or variants have been actively circulating in Western Sudan, yet undetected for decades. In fact, sporadic cases and outbreaks of acute febrile hemorrhagic illnesses compatible with CCHF have long been observed in Kordufan region for several past decades, but the virus was not identified until very recently [4]. Given the fact that most of CCHF sporadic cases and outbreaks occur in villages and remote areas, together with combined unfamiliarity of the Sudanese physicians with clinical signs and symptoms of CCHFV infections, it is most likely that CCHF cases can easily pass unreported or misdiagnosed. Currently, CCHF is not considered in the differential diagnosis of acute hemorrhagic illness in Sudan.

In Sudan, fever of unknown etiology is not uncommon, but malaria is usually considered to be the primary cause [19]. The first line of treatment in the hospitals focused primarily on prescription of anti-malarial medication but without improvement. Findings of this study illustrate that malaria positive result does not necessarily eliminates the possibility of co-infection with other viral hemorrhagic fever agents. Interestingly enough, both patients were malaria positive as is the case with Alfulah and Abyei CCHF outbreaks [4,5]. Currently we do not have an explanation for the association of malaria and CCHF positive cases. Therefore, physician should consider CCHF in their differential diagnosis when dealing with malaria cases in CCHF areas of endemicity.

In the present study, we confirmed that cluster of two cases was due to CCHFV as determined by RT-PCR and serology. Clearly, the disease is becoming endemic in most areas of Kordufan region of Sudan. The two CCHFV strains involved are identical and belonged to group III virus lineage as determined by the S segment partial sequences and subsequent phylogenetic analysis [16]. The name Lagawa strain was initially proposed for this CCHFV strain. The partial S segment sequence of Lagawa strain, described in this study, shows 99% homology to Alfulah strain (GenBank accession number GQ862371) previously described by Aradaib et al., [4]. It is suggested that whole genome of this CCHFV strain be sequenced to obtain better information on the virus and the possibility of genetic reassortment and/or recombination events [8,20,21].

The CCHFV is susceptible to ribavirin in vitro but there is no controlled study evaluating oral versus intravenous ribavirin in treating infected patients. However, few studies have evaluated oral ribavirin. Oral ribavirin, at a dose rate of 200 mg twice daily for 5 days, is the recommended dosage for post-exposure prophylaxis [22]. In the present study, prescription of oral ribavirin, at a dose rate of 500 mg 4 times a day for 5 days, and administration of platelets concentrate resulted in rapid improvement of the health condition of the physician. Intravenous compensation with blood, platelets concentrate and fluid therapy (dextrose and saline), augmented oral ribavirin therapy as demonstrated by dramatic increase in blood platelets counts. The treatment resulted in improved health condition of the female physician.

In conclusion, this study highlights the need to include CCHF in the differential diagnosis, when fever with hemorrhagic tendencies is observed in clinical centers and hospitals in the Kordufan region, Sudan. The frequent occurrence of sporadic cases and multiple CCHF outbreaks, and the risks these cases pose for medical staff in resource-poor health care facilities, necessitates the need for improved surveillance programs and prevention measures for this important viral disease in Sudan.

## Competing interests

The authors declare that they have no competing interests.

## Authors' contributions

ATME optimized the nested polymerase chain reaction-based detection assay; MSK designed the study and prepared the draft manuscript; RME processed the clinical samples and perform the viral RNA extraction; MAH conducted examination of clinical cases and clinical presentation of CCHF cases; KHT preparation and editing of nucleic acid sequences of the virus strain; MIE designed the experiment; IEA designed the experiment and prepared the final manuscript. All authors read and approved the final manuscript.
